# Efficacy and Tooth Sensitivity of Low‐ Versus High‐Concentration Hydrogen Peroxide for In‐Office Bleaching: A Randomized Clinical Trial

**DOI:** 10.1111/jerd.70090

**Published:** 2025-12-29

**Authors:** Gabrielle Gomes Centenaro, Michael Willian Favoreto, Taynara de Souza Carneiro, Deisy Cristina Ferreira Cordeiro, Alessandro D. Loguercio

**Affiliations:** ^1^ Department of Restorative Dentistry State University of Ponta Grossa Paraná Brazil; ^2^ Department of Restorative Dentistry Tuiuti University of Paraná Paraná Brazil; ^3^ Area of Stomatology, IDIBO Research Group, Health Sciences Faculty Rey Juan Carlos University Madrid Spain

**Keywords:** bleaching agents, clinical trial, color bleaching, hydrogen peroxide, tooth bleaching, tooth sensitivity

## Abstract

**Objective:**

This randomized, parallel, double‐blind clinical trial aimed to evaluate the equivalence in bleaching efficacy (BE), as well as the risk and intensity of tooth sensitivity (TS), in participants undergoing in‐office dental bleaching with low (6%) versus high‐concentration (35%) hydrogen peroxide.

**Material and Methods:**

A total of 140 participants were randomly assigned to one of the two groups according to the bleaching gel concentration used: 6% hydrogen peroxide (Automixx 6%, FGM) or 35% hydrogen peroxide (Automixx Plus 35%, FGM), the bleaching procedure was performed for 50 min in three sessions, with 1‐week interval. The bleaching procedure was performed for 50 min in three sessions, with a 1‐week interval. BE was evaluated using a Vita Easyshade spectrophotometer (ΔE_00_, ΔE_ab_, and ΔWI_D_) and both Vita Classical and Vita Bleachedguide shade guides (ΔSGU) at days 7, 14, and 21, and 1 month after completion treatment. The risk and intensity of TS were recorded using a visual analog scale (VAS; 0 to 10), assessed immediately after each of the three bleaching sessions, and at 1, 24, and 48 h posttreatment. BE was assessed with a paired Student's *t* test. The absolute risk of TS was evaluated using the Fisher's exact test, and the TS intensity was assessed using a paired Student's *t* test (α = 0.05).

**Results:**

Nonequivalent bleaching outcomes were observed between the groups across all instruments and time points (*p* > 0.05). The 35% hydrogen peroxide group showed significantly greater efficacy than the 6% group (MD in ΔE_00_ = 6.3; 90% CI 4.8–7.7; *p* < 0.05). The risk of TS was lower in the 6% group (44%) compared with the 35% group (74%; *p* = 0.0005). Sensitivity intensity was also significantly lower in the 6% group up to 24 h posttreatment (*p* < 0.01).

**Conclusions:**

In‐office dental bleaching with 6% and 35% hydrogen peroxide resulted in significant tooth whitening; however, the outcomes were not equivalent across all evaluation time points, with the 35% concentration demonstrating superior efficacy throughout the study. In contrast, TS was significantly higher in the 35% group compared with the 6% group.

**Clinical Relevance:**

While 35% hydrogen peroxide delivers more effective tooth whitening in‐office, it is also associated with a higher risk and intensity of TS. Lower concentrations like 6% offer a safer alternative with reduced sensitivity but less whitening efficacy.

## 1 Introduction

Tooth bleaching has become increasingly relevant in daily dental practice due to the growing aesthetic demands from patients [1]. This trend reflects widespread dissatisfaction with tooth color [1, 2], often amplified by social media influences and their impact on self‐esteem, where constant comparisons with others are common [3, 4].

Two well‐established tooth bleaching techniques are frequently discussed in the literature: at‐home and in‐office treatments [5, 6]. Although both methods demonstrate comparable bleaching efficacy, in‐office bleaching is often favored for delivering faster results, right after the first session [5, 6, 7, 8]. This accelerated outcome is largely attributed to the use of high concentrations of hydrogen peroxide, typically ranging from 35% to 40% [5, 9, 10].

However, the primary adverse effect reported by patients undergoing in‐office bleaching is procedure‐induced tooth sensitivity (TS) [6, 9, 11, 12]. This TS arises from the diffusion of hydrogen peroxide through the dental structure, penetrating the enamel and dentin to reach the pulp chamber, where it can trigger inflammatory responses [13, 14, 15]. The clinical signs associated with TS are characterized by a sharp, short‐lasting pain that tends to intensify within the first hour after the bleaching session and typically resolves spontaneously within 48 h [16, 17, 18]. Recent randomized clinical trials using high concentrations of hydrogen peroxide have reported TS in approximately 80% of participants [16, 17, 18].

In light of this, several strategies are being explored to reduce or prevent adverse effects. These include reducing the application time of the bleaching gel [17, 19, 20], using more alkaline and chemically stable bleaching gels rather than acidic formulations [21, 22], and adjusting the volume of bleaching gel applied [16, 23], as this influence hydrogen peroxide diffusion into the pulp chamber [24].

Moreover, reducing the concentration of in‐office bleaching agents has also been shown to decrease the incidence of TS [8, 25], as lower amounts of hydrogen peroxide are available to penetrate the pulp chamber. Therefore, lowering the concentration of the bleaching gel represents a promising strategy for reducing bleaching‐induced TS.

Indeed, this concept is not new, as two systematic reviews of randomized clinical studies have confirmed that lower concentrations of hydrogen peroxide are associated with reduced bleaching‐induced TS [8, 25]. However, the evidence regarding bleaching efficacy remains controversial. For instance, while some studies have shown that hydrogen peroxide concentrations below 30% result in inferior bleaching efficacy compared to higher concentrations [25], others have reported comparable or even superior outcomes using lower concentrations (typically ranging from 6% to 20%).

Notably, the definition of “lower concentration” varies considerably across the literature. However, following recent European regulations that limit the maximum allowable concentration of hydrogen peroxide to 6% for both in‐office and at‐home bleaching procedures, this specific concentration has received increasing attention in clinical research [26].

Although the use of 6% hydrogen peroxide is not new, several randomized clinical studies have investigated this specific concentration [23, 27, 28, 29, 30] and reported promising outcomes regarding reduced TS. However, conflicting findings persist among studies comparing 6% hydrogen peroxide to higher concentrations, such as 35%–37.5% hydrogen peroxide [27, 28, 29, 30]. While some trials have demonstrated comparable bleaching efficacy between these concentrations [27, 28], others have reported significantly lower bleaching efficacy for the 6% formulation [29, 30]. Further well‐designed randomized clinical trials directly comparing 6% and 35% hydrogen peroxide are warranted to clarify these discrepancies in bleaching performance.

Therefore, the aim of this equivalence, parallel, randomized, double‐blind clinical trial was to evaluate the bleaching efficacy, as well as the risk and the intensity of TS, of high‐ and low‐concentration hydrogen peroxide gels in a self‐mixed system, a different delivery method that enables the clinician to prepare the mixture immediately before application. In addition, the bleaching gels evaluated in this study maintain a neutral to slightly alkaline pH throughout the entire procedure (ranging between 7 and 8) [10, 31]. The research hypotheses tested were that (1) the bleaching efficacy of the two concentrations (6% vs. 35%) would be equivalent within the predefined clinical margins for all evaluated color parameters, and (2) the intensity and risk of TS induced by in‐office bleaching would differ in the risk and intensity of TS between the two concentrations.

## 2 Materials and Methods

### 2.1 Study Design

This study was submitted to and approved by the Ethics Committee of the State University of Ponta Grossa (Ponta Grossa/PR/Brazil) and was prospectively registered in the Brazilian Registry of Clinical Trials (RBR‐275z28y). The study adhered to the Consolidated Standards of Reporting Trials (CONSORT) guidelines [32] and the extension for equivalence studies [33]. An equivalence‐based, parallel, double‐blind, randomized controlled design with an equal allocation ratio was employed. The trial was conducted from February 2025 to June 2025 at the clinics of the School of Dentistry at the State University of Ponta Grossa.

### 2.2 Recruitment

Participants were recruited via written advertisements posted on university premises and through social media, forming a convenience sample. They were informed of the study objectives, and all participants provided written informed consent prior to enrollment.

### 2.3 Eligibility Criteria

To be eligible for this randomized clinical trial, participants were required to be at least 18 years old, in good general and oral health, and to have no history of TS. All six maxillary anterior teeth had to be free of carious lesions, restorations, and periodontal disease, with canines classified as shade A2 or darker, based on a value‐oriented shade guide unit (Vita Classical A1‐D4 shade guide, Vita Zahnfabrik, Bad Säckingen, Germany).

Exclusion criteria included individuals with dental prostheses, orthodontic appliances, or significant internal tooth discoloration (e.g., tetracycline stains, fluorosis, or nonvital teeth). Pregnant or lactating women, individuals with bruxism, users of analgesics or anti‐inflammatory medications, those with prior tooth‐bleaching treatments, or those presenting any condition potentially causing sensitivity (e.g., gingival recession, dentin exposure, or visible enamel cracks) were also excluded.

### 2.4 Sample Size Estimation

For this randomized clinical trial, the primary outcome considered for sample size estimation was the objective color change measured by ΔE_00_. In a previous study, a ΔE_00_ value of 8.3 ± 2.0 was observed after treatment with 35% hydrogen peroxide gel, the same product used in the present study [16]. Using a 50%:50% perceptible threshold of 1.2 [33], it was determined that 61 volunteers per group would be necessary to detect no significant difference between the standard (35% hydrogen peroxide) and experimental (6% hydrogen peroxide) treatments. This calculation provides 90% power to detect, at a 5% significant level, a difference in means no greater than 1.2 (www.sealedenvelope.com). To account for potential losses, an additional 15% was added, resulting in a total sample size of 70 volunteers per group, or 140 volunteers in total.

### 2.5 Random Sequence Generation and Allocation Concealment

Randomization was performed using freely available software (www.sealedenvelope.com) with block sizes of 4, 6, and 8 to ensure an equal allocation ratio. Participants were randomly assigned to two groups (*n* = 70 each) according to the concentration of the in‐office bleaching gel. One group received a 6% hydrogen peroxide gel (Automixx 6%, FGM Dental Group, Joinville, SC, Brazil), and the other group received a 35% hydrogen peroxide gel (Automixx Plus 35%, FGM Dental Group, Joinville, SC, Brazil). The allocation sequence was concealed using opaque, sealed, and sequentially numbered envelopes. The researcher responsible for randomization and blinding was not involved in the intervention process. Before each bleaching procedure, the operator opened the envelope to determine each participant's assigned group.

### 2.6 Blinding

This study was double‐blind, meaning that both the evaluator and the participant were unaware of group assignments. However, due to the differing concentrations of bleaching gel (6% vs. 35%) used in the bleaching procedure, the operator was necessarily aware of the assigned treatment throughout the study.

### 2.7 Study Intervention

Two weeks prior to the in‐office dental bleaching, prophylaxis was performed on all 140 participants to remove extrinsic stains. This procedure involved using pumice stone (Biodinâmica, Ibiporã, PR, Brazil) and water with a rubber cup attached to a contra‐angle handpiece (Preven, Guapirama, PR, Brazil), along with oral hygiene instructions.

To begin the bleaching procedure, an ArcFlex retractor (FGM Dental Group, Joinville, SC, Brazil) was placed, and the gingiva was protected by applying a light‐curing resin barrier (Topdam, FGM Dental Group, Joinville, SC, Brazil). The operator then opened the envelope to reveal each participant's assigned bleaching treatment, either 6% or 35% hydrogen peroxide.

Following randomization, the assigned bleaching gel (6% or 35%) was applied in a single 50‐min session according to the manufacturer's instructions, without renewal. Subsequently, the gel was removed using a disposable suction device, and the teeth were cleaned with gauze and thoroughly rinsed with water. The bleaching procedure was identical for all participants, with a 1‐week interval between each session, resulting in three bleaching sessions per treatment.

### 2.8 Color Evaluation

All color parameters were recorded prior to the bleaching procedure, as well as after the first, second, and third sessions, and 30 days after treatment completion. Each session was evaluated at the subsequent appointment to prevent temporary tooth dehydration and demineralization from affecting the color assessment. Two blinded and calibrated examiners performed the measurements in a room with controlled artificial lighting to prevent any interference from natural light. During assessments, all participants remained seated, and the middle third of the right maxillary and mandibular canines was used as the tooth‐matching area [17, 18, 34].

#### 2.8.1 Objective Color Evaluation

The Vita Easyshade spectrophotometer (VITA Easyshade Advance 4.0, Vita Zahnfabrik, Bad Säckingen, Germany) was used following the CIEL*a*b* parameters [35, 36, 37]. In this system, L* represents the lightness (ranging from 0 = black to 100 = white), while a* and b* represent chromaticity coordinates with a* measuring the red‐green axis and b* the yellow‐blue axis. All color measurements were performed in a room with controlled artificial lighting to eliminate any interference from natural light. To standardize color measurement, an impression of the maxillary arch was obtained using green dense silicone paste (Perfil, Coltene, Rio de Janeiro, RJ, Brazil). Considering that the color of the silicone can influence the color measurement [38], the same silicone color was used throughout the entire experiment. A 6 mm‐diameter window was created using a metal device (Biopsy Punch, Miltex, York, NJ, USA), matching the diameter of the spectrophotometer tip. The tip was inserted into the silicone guide to obtain the L*, a*, and b* values.

#### 2.8.2 Bleaching Efficacy (Color Change by ΔE_00_ and ΔE_ab_ )

To determine the perceptible and clinical color change between baseline and follow‐up intervals, the CIEDE2000 color difference formula (ΔE_00_) was used [39]. Color changes were considered clinically perceptible when ΔE_00_ exceeded 0.8, based on the 50:50 perceptibility threshold, where 50% of observers would notice a difference under standardized conditions [33].

To determine the perceptible and clinically relevant color change between baseline and follow‐up intervals, the CIEL*a*b* color difference formula (ΔE_ab_) was used [40]. Color changes were considered clinically perceptible when ΔE_ab_ exceeded 1.2, based on the 50:50 perceptibility threshold, where 50% of observers would notice a difference under standardized conditions [41].

#### 2.8.3 Whiteness Assessment (Whiteness Index for Dentistry—WI_D_ )

To calculate the WI_D_, the following formula was applied: [42] WI_D_ = 0.551 × L—2.324 × a—1.1 × b. This index was used to determine the baseline color and follow‐up intervals for each specimen. This index quantifies the perceived degree of tooth whiteness, independent of general color change. Differences in WI_D_ (ΔWI_D_) between baseline and follow‐up were used to evaluate whitening intensity. A ΔWI_D_ value greater than 0.6 units was considered clinically perceptible, also based on the 50:50% perceptibility threshold [43]. Values below this threshold were interpreted as clinically insignificant [43].

#### 2.8.4 Subjective Color Evaluation

The value‐oriented Vita Classical shade guide (Vita Zahnfabrik, Bad Säckingen, Germany), consisting of 16 color tabs arranged from the highest value (B1) to lowest (C4) [17, 18, 34, 43], was used. Additionally, the Vita Bleachedguide 3D‐MASTER scale (Vita Zahnfabrik, Bad Säckingen, Germany), specifically designed for tooth bleaching evaluations due to its inclusion of lighter color tabs, was employed. This scale ranges from highest value (0 M1) to the lowest (5 M3) [17, 18, 34, 43]. Color change in shade guide units (ΔSGU) for the middle third of the vestibular surface of the right canine was calculated by subtracting the baseline shade guide number from the final shade guide number, both organized by value.

### 2.9 Assessment of Tooth Sensitivity

To assess the intensity and absolute risk of TS, the Visual Analogue Scale (VAS; 0–10) was used, where 0 indicated no pain and 10 represented severe pain. Participants received a TS diary for each bleaching session [11, 17, 18, 21, 44, 45, 46]. They were instructed to record the highest level of pain at the following time points: (1) immediately after the bleaching session; (2) up to 1 h after; (3) up to 24 h after; and (4) up to 48 h after the bleaching session. Participants were asked to mark a vertical line on the scale corresponding to their perceived TS intensity, which was later measured in centimeters—even if no pain was experienced. Any score above zero was considered indicative of TS resulting from the bleaching procedure, while a score of zero indicated no TS. Clear guidelines were provided to ensure accurate reporting. This process was repeated across all three bleaching sessions, and the highest score recorded per participant was used for statistical analysis.

### 2.10 Statistical Analysis

The statistician responsible for data analysis was blinded to group assignments. In cases of missing data, referring to participants who did not attend the scheduled color assessment appointments, the last observation was carried forward.

For all outcomes, statistical analyses adhered to the intention‐to‐treat (ITT) protocol, including all participants who began the trial. Equivalence testing was performed for the primary outcome ∆E_00_ value and all other color parameters (∆E_ab_, ∆WI_D_, and ΔSGUs) 1 month postbleaching. Using the Two One‐Sided Tests (TOST) approach, equivalence was assessed by performing two one‐tailed *t* tests: one to test if the mean difference exceeded the lower equivalence limit (−Δ) and another to test if it was below the upper equivalence limit (+Δ). Equivalence was concluded if both tests rejected the research hypotheses of nonequivalence, indicating that the observed mean difference lay entirely within the predefined equivalence margins. The larger *p* value from the two tests was reported.

Predefined equivalence thresholds were based on 50:50 perceptibility thresholds previously reported in the literature (∆E_00_ [< −0.8 or > 0.8], ∆E_ab_ [< −1.2 or > 1.2], and ∆WI_D_ [< −0.6 or > 0.6]) [33, 43] ΔSGU VITA classical (post hoc threshold of < −1 or > 1 unit) and ΔSGU Vita Bleachedguide (post hoc threshold of < −2 or > 2 units). Additional analyses were performed for earlier time points (baseline vs. 1 week, vs. 2 weeks, and vs. 3 weeks). If equivalence was not determined, post hoc *t* tests were employed to explore differences between groups at each assessment period.

The absolute risk of TS was compared using Fisher's exact test, and the odds ratio with the corresponding confidence interval (CI) was calculated. The intensity of TS was analyzed using a Student's *t* test. All statistical tests were conducted at a 5% significance level using the SigmaPlot software (Version 11.0, Systat Software, San Jose, CA, USA).

## 3 Results

### 3.1 Characteristics of Included Participants

**FIGURE 1:**
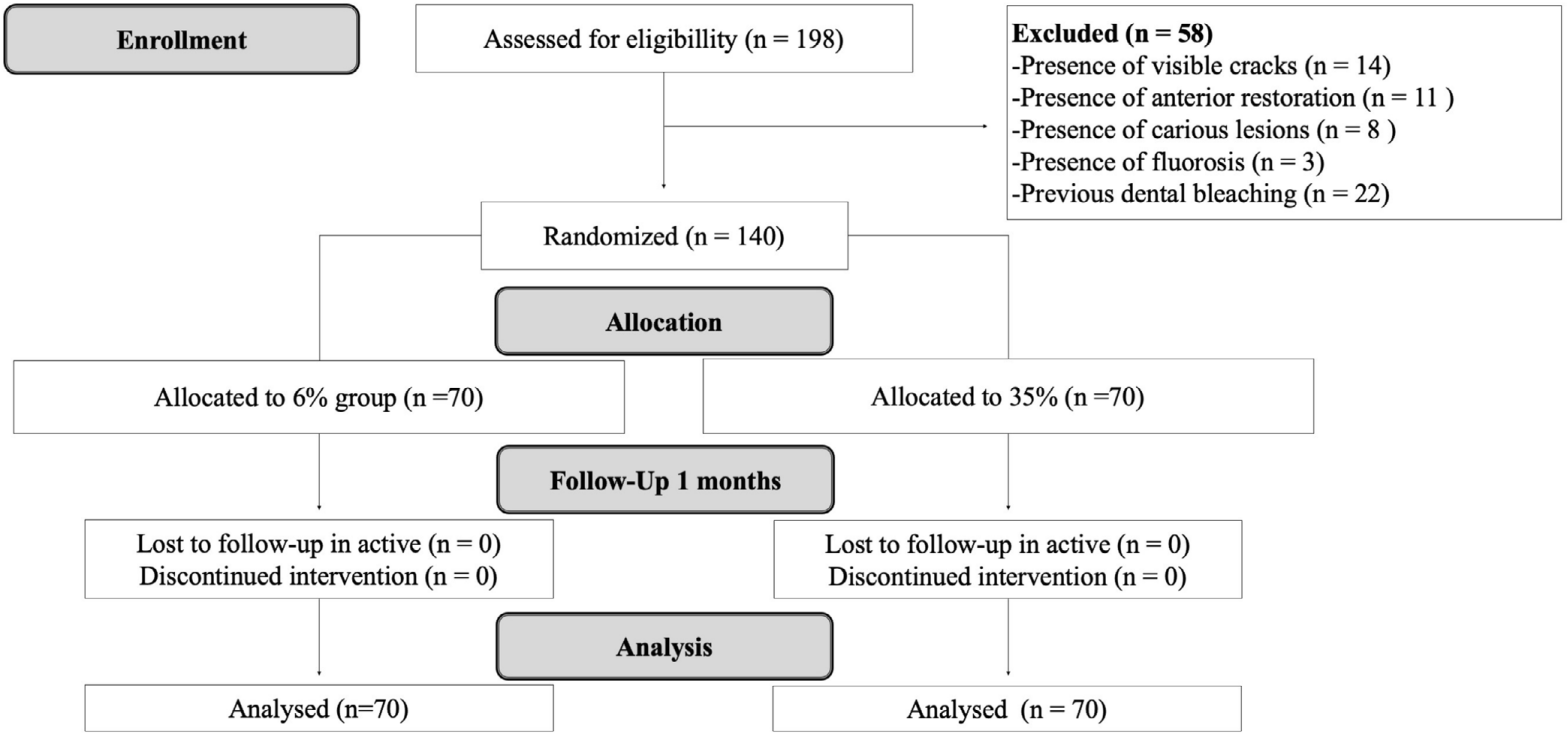
Flow diagram of the clinical trial including detailed information on the excluded participants.

A total of 198 participants were initially examined, of whom 140 were included in this randomized clinical study. No participants were lost to follow‐up during the study period (Figure 1). Table 1 presents the baseline tooth color and the distribution of participants by gender and age.

**TABLE 1:** Baseline characteristics of the participants.

	6% hydrogen peroxide	35% hydrogen peroxide
	(*n* = 70)	(*n* = 70)
Baseline color (SGU vita classical; mean ± SD)	9.3 ± 2.6	9.6 ± 2.3
Baseline color (SGU vita bleachedguide; mean ± SD)	18.7 ± 2.5	19.5 ± 2.2
Baseline color (WI_D_; mean ± SD)	16.2 ± 7.4	17.1 ± 5.3
Age (years; mean ± SD)	23.6 ± 2.8	23.8 ± 3.7
Gender (female; %)	65	62

Abbreviations: SGU, shade guide unit measured by scale color evaluation; WI_D_, Whiteness Index for Dentistry baseline measured by Vita Easy Shade spectrophotometer.

### 3.2 Bleaching Efficacy

**FIGURE 2:**
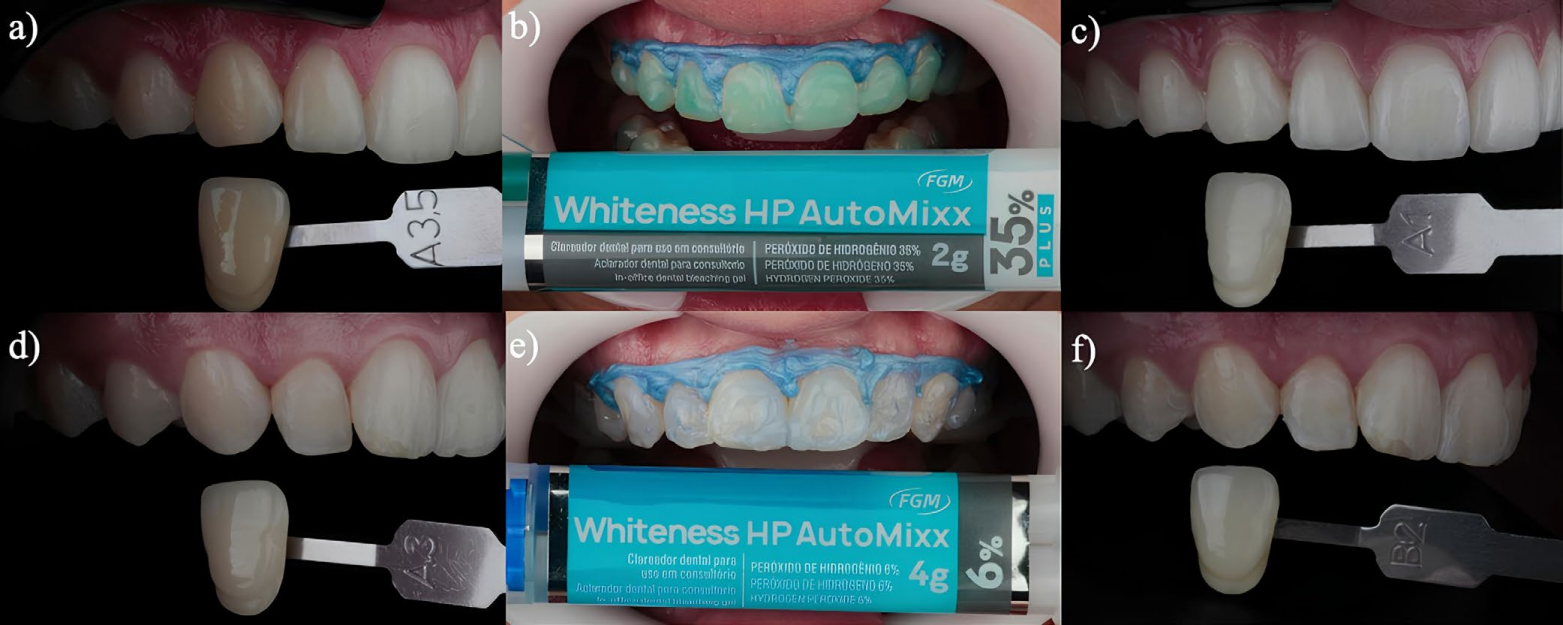
(a) Initial color measurement performed using Vita Classical shade guide A1‐C4. (b) Bleaching session with 35% hydrogen peroxide. (c) Final color measurement. (d) Initial color measurement performed using Vita Classical shade guide A1‐C4. (e) Bleaching session with 6% hydrogen peroxide. (f) Final color measurement.

The results of bleaching efficacy are summarized in Table 2. Both bleaching gels demonstrated a significant increase in whitening (Figure 2), as measured by both objective and subjective color assessment methods. This is evident from the fact that, for both groups, the whitening observed at all assessment times exceeded the thresholds for visually perceptible differences: 0.8 for ΔE_00_, 1.2 for ΔE_ab_, 0.6 for ΔWI_D_, 1.0 for ΔSGU VITA Classical, and 2.0 for ΔSGU VITA Bleachedguide. One month after the bleaching procedure, the equivalence of color changes between the PH 6% and PH 35% groups was assessed. For ΔE_00_, the mean difference (MD, calculated as PH35%–PH6%) was 1.6 (90% CI 0.7–2.4), *p* = 1.000, which lies entirely outside the clinically perceptibility equivalence margins of −0.8 to +0.8. Similarly, for ΔE_ab_, the MD was 2.5 (90% CI 1.4–3.7), *p* = 1.000, which also exceeded the equivalence margins of −1.2 to +1.2. For ΔWI_D_, the MD was 9.7 (90% CI 7.5–12.0), *p* = 1.000, falling outside the predefined equivalence range of −0.6 to +0.6. For visual color changes measured in ΔSGU, the results were consistent with the instrumental data. For the VITA Classical shade guide, the MD was 4.4 (90% CI 3.7–5.0), *p* = 1.000, and for the VITA Bleachedguide shade guide, the MD was 6.6 (90% CI 5.9–7.3), *p* = 1.000, both of which lie outside the equivalence thresholds of −2.0 to +2.0 (Table 2).

**TABLE 2:** Means, standard deviations, and mean difference for both study groups obtained with the different color assessment tools at the different evaluation periods.

Periods	Group				
	6% hydrogen peroxide	35% hydrogen peroxide	MD	*p*	
	Mean ± SD	Mean ± SD	(90% CI)	Equivalence^a^	Difference^b^
	ΔE_00_, equivalence margin ± 0.8				
Baseline vs.					
1 week	5.4 ± 4.4	6.4 ± 2.7	1.0 (0.1 to −2.0)	1.000	0.124
2 weeks	5.4 ± 3.5	8.1 ± 2.5	2.6 (1.8–3.5)	1.000	< 0.001
3 weeks	6.4 ± 3.8	8.1 ± 2.7	1.7 (0.8–2.6)	1.000	0.003
1 month	6.2 ± 3.5	7.8 ± 2.5	1.6 (0.7–2.4)	1.000	0.003
	ΔE_ab_, equivalence margin ± 1.2				
1 week	7.8 ± 5.9	9.8 ± 4.2	2.0 (0.6–3.5)	1.000	0.020
2 weeks	8.0 ± 4.6	12.4 ± 3.3	4.4 (3.3–5.6)	1.000	< 0.001
3 weeks	9.6 ± 5.1	12.5 ± 3.6	2.9 (1.7–4.2)	1.000	< 0.001
1 month	9.3 ± 4.8	11.8 ± 3.4	2.5 (1.4–3.7)	1.000	< 0.001
	ΔWI_D_, equivalence margin ± 0.6				
1 week	4.8 ± 10.0	13.9 ± 6.6	9.1 (6.7–11.5)	1.000	< 0.001
2 weeks	8.2 ± 9.5	18.5 ± 4.9	10.2 (8.1–12.4)	1.000	< 0.001
3 weeks	6.9 ± 9.3	18.4 ± 5.4	11.5 (9.4–13.6)	1.000	< 0.001
1 month	7.9 ± 10.0	17.6 ± 5.2	9.7 (7.5–12.0)	1.000	< 0.001
	ΔSGU VITA classical, equivalence margin ± 1.0				
1 week	0.7 ± 1.3	5.7 ± 2.2	5.0 (4.5–5.5)	1.000	< 0.001
2 weeks	2.0 ± 1.7	7.6 ± 2.2	5.6 (5.1–6.2)	1.000	< 0.001
3 weeks	3.2 ± 2.1	7.6 ± 2.2	4.4 (3.8–5.0)	1.000	< 0.001
1 month	3.3 ± 2.2	7.7 ± 2.3	4.4 (3.7–5.0)	1.000	< 0.001
	ΔSGU VITA Bleachedguide, equivalence margin ± 2.0				
1 week	0.7 ± 1.4	6.5 ± 2.6	5.8 (5.2–6.4)	1.000	< 0.001
2 weeks	2.2 ± 1.7	10.1 ± 2.7	7.9 (7.2–8.5)	1.000	< 0.001
3 weeks	3.5 ± 2.1	10.1 ± 2.7	6.6 (5.9–7.3)	1.000	< 0.001
1 month	3.4 ± 2.1	10.0 ± 2.7	6.6 (5.9–7.3)	1.000	< 0.001

*Note*: The equivalence margins for ΔE_ab_, ΔE_00_, and ΔWI_D_ corresponds to the 50:50 perceptibility threshold for each color change parameter.

Abbreviations: CI, confidence interval; MD, mean difference; SGU, shade guide units; WI_D_, Whiteness Index for Dentistry.

^a^ *p* value for equivalence using TOST methodology; that is *p* < 0.05 implies that the two groups had equivalent means.

^b^ *p* value for difference using null‐hypothesis significance testing; that is, *p* < 0.05 implies that the two groups had different means.

In post hoc analyses, in terms of objective evaluation, statistically significant differences were observed between the two groups for all color evaluation parameters 1 month after treatment (ΔE_00_; *p* = 0.003; ΔE_ab_; *p* < 0.001; ΔWI_D_; *p* < 0.001), with the 35% hydrogen peroxide demonstrating superior outcomes. According to the subjective assessment scales (Table 2), significant differences in bleaching efficacy were also found in favor of the 35% hydrogen peroxide group after 1 month (*p* < 0.05; Table 2). Specially, both subjective scales showed statistically significant differences between the groups (Vita Classical; *p* < 0.001; Vita Bleachedguide; *p* < 0.001).

### 3.3 Tooth Sensitivity (TS)

The absolute risk of TS was 44% in the 6% hydrogen peroxide group and 74% in the 35% hydrogen peroxide group, resulting in a relative risk of 0.59 (95% CI = 0.44–0.80; Table 3). This indicates that the 6% hydrogen peroxide group showed a significantly lower absolute risk of TS compared to the 35% hydrogen peroxide group (Table 3; *p* = 0.0005).

**TABLE 3:** Absolute risk of tooth sensitivity (TS) in both study groups, along with relative risk and 95% confidence interval (CI).

Protocols	Number of participants with TS		Absolute risk (95% CI)	Relative risk^a^ (95% CI)
	Yes	No		
6% hydrogen peroxide	31	39	44 (33–55)	0.59 (0.44–0.80)
35% hydrogen peroxide	52	18	74 (62–83)	

^a^ Fisher's exact test (*p* = 0.0005).

Regarding the intensity of TS, statistically significant differences were observed between the two groups across all three bleaching sessions (Table 4). Specifically, significant differences were found in the immediate posttreatment evaluation (*p* < 0.002), as well as up to 1 h (*p* < 0.001) and 24 h (*p* < 0.001). However, no significant differences were observed at 48 h (*p* > 0.09; Table 4). Overall, lower sensitivity intensity values were consistently recorded in the 6% hydrogen peroxide group compared to the 35% hydrogen peroxide group (Table 4; *p* < 0.01).

**TABLE 4:** Means and standard deviations of tooth sensitivity intensity (VAS) for both study groups at the different assessment periods, including the worst score^a^ reported by participants across the bleaching sessions.

	Periods	6% hydrogen peroxide	35% hydrogen peroxide	MD (95% CI)	*p* ^b^
First bleaching session	Immediate	0.2 ± 0.8	1.0 ± 1.7	0.8 (0.83–1.2)	< 0.01
	Until 1 h	0.2 ± 0.9	1.5 ± 2.0	1.2 (0.7–1.7)	< 0.01
	Until 24 h	0.1 ± 0.4	0.5 ± 1.0	0.4 (0.2–0.7)	< 0.001
	Until 48 h	0.0 ± 0.3	0.1 ± 0.4	0.1 (− 0.1 to 0.2)	0.340
Second bleaching session	Immediate	0.1 ± 0.1	0.8 ± 1.7	0.7 (0.3–1.1)	< 0.002
	Until 1 h	0.1 ± 0.1	1.5 ± 2.3	1.4 (0.9–2.0)	< 0.01
	Until 24 h	0.1 ± 0.0	1.2 ± 2.0	1.1 (0.6–1.6)	< 0.01
	Until 48 h	0.0 ± 0.0	0.2 ± 0.9	0.2 (− 0.03 to 0.4)	0.093
Third bleaching session	Immediate	0.1 ± 0.1	0.9 ± 1.7	0.7 (0.3–1.1)	< 0.01
	Until 1 h	0.1 ± 0.0	1.5 ± 2.2	1.5 (0.9–2.0)	< 0.01
	Until 24 h	0.1 ± 0.0	1.2 ± 2.0	1.1 (0.7–1.6)	< 0.01
	Until 48 h	0.0 ± 0.0	0.2 ± 0.9	0.2 (− 0.03 to 0.4)	0.093
Worst score from sessions		0.4 ± 1.0	2.9 ± 2.7	2.5 (1.8–3.2)	< 0.01

Abbreviation: MD, mean difference.

^a^ The worst score from both sessions refers to the highest tooth sensitivity score reported by each participant during the three bleaching sessions.

^b^ Student's *t* test for independent samples.

## 4 Discussion

Tooth bleaching is influenced by the concentration of the bleaching gel applied to the enamel surface, as well as its efficacy and potential side effects, such as TS [5, 8]. To the best of the authors' knowledge, previous randomized clinical trials directly comparing high and low concentrations of hydrogen peroxide for in‐office dental bleaching were conducted with relatively small sample sizes (approximately 30–33 participants per study), which nonetheless yielded consistent and valid findings [27, 28, 29, 30]. In contrast, the present trial included a substantially larger sample per group, thereby enhancing the robustness and external validity of the results when comparing 6% and 35% hydrogen peroxide self‐mixing formulations for in‐office use. Despite these methodological improvements, the most relevant contribution of the present study is that it aimed to address the conflicting findings reported in previous studies comparing 6% hydrogen peroxide with higher concentrations, such as 35%–37.5% hydrogen peroxide [27, 28, 29, 30].

Bleaching efficacy was observed in both groups treated with different concentrations of hydrogen peroxide gel compared to baseline. This outcome is supported by the fact that the color change values in both groups exceeded the perceptibility thresholds for all evaluation methods (ΔE_00_, ΔE_ab_, and ΔWI_D_), indicating that both concentrations produced clinically perceptible whitening effects. For the objective color evaluation methods (ΔE_00_, ΔE_ab_, and ΔWI_D_), the 50%:50% perceptibility thresholds were applied: ΔE_00_ (0.8), ΔE_ab_ (1.2) [42], and ΔWI_D_ (0.6) [43]. These thresholds represent the point at which 50% of observers would consider the color difference perceptible in a clinical context, making them useful benchmarks for evaluating the perceptibility of color changes in dentistry.

The study showed that the bleaching efficacy was not equivalent between the groups for any of the color assessment instruments, rejecting the first research hypothesis. This means that the mean differences and their respective 90% CIs exceeded the pre‐established equivalence margins. However, the equivalence test does not determine whether there is a statistically significant difference between the groups or which group performs better. Therefore, post hoc tests were conducted to explore multiple comparisons among the groups. At all evaluation time points and across all color assessment methods, both objective and subjective, the 35% hydrogen peroxide concentration consistently outperformed the 6% hydrogen peroxide concentration. This can be explained by the fact that, at higher concentrations, a greater amount of hydrogen peroxide penetrates the enamel and dentin more rapidly, releasing a higher volume of free radicals, such as hydroxyl and perhydroxyl ions [34]. These radicals are primarily responsible for breaking down pigmented molecules within the tooth structure. As a result, more extensive oxidation of chromogens occurs [37], leading to visibly greater changes in tooth color compared to lower concentrations of hydrogen peroxide. These results are consistent with previous randomized clinical trials that compared 35% hydrogen peroxide with 6% [29, 30].

However, some clinical trials have shown that 6% hydrogen peroxide can achieve bleaching efficacy comparable to that of higher concentrations [27, 28]. This effect is attributed to the combination of the low‐concentration gel with nitrogen‐doped titanium dioxide (N‐TiO_2_), which is activated by a hybrid light source. The photocatalytic action of N‐TiO_2_ enhances the decomposition of hydrogen peroxide [35], generating reactive oxygen species that accelerate the whitening process and compensate for the lower peroxide content [27, 28]. Therefore, the synergistic interaction between the low‐concentration hydrogen peroxide and the light‐activated titanium dioxide allowed the 6% gel to achieve effective bleaching outcomes comparable to those of the more concentrated 35% gel [27, 28]. Interesting, however, a recent clinical trial found no significant improvement in bleaching efficacy when 6% hydrogen peroxide containing N‐TiO_2_ was activated by light compared to when it was not [46]. These findings highlight the need for further clinical research to clarify the role of light activation in enhancing the efficacy of low‐concentration hydrogen peroxide gels containing N‐TiO_2_. However, since the present study did not use any bleaching gel containing TiO_2_, this mechanism does not apply to our results.

It is important to note that the differences between the two groups were consistent across all objective color evaluation methods, based on the 50%:50% perceptibility thresholds: ΔE_00_ = 0.8, ΔE_ab_ = 1.2 [42], and ΔWI_D_ = 0.6 [43]. When applying the ΔWI_D_ formula, the 50%:50% perceptibility threshold (0.6) was exceeded at all assessment time points, as were ΔE_00_ and ΔE_ab_. Notably, ΔWI_D_ values indicated an overall increase in whitening for both hydrogen peroxide concentrations throughout the study, with the high‐concentration group consistently exhibiting higher ΔWI_D_ values than the low‐concentration group at all assessment time points.

Regarding the risk and intensity of TS, the use of a bleaching gel with a lower hydrogen peroxide concentration reduced the likelihood of this adverse effect by 30% compared to the more concentrated gel typically used for in‐office bleaching. Additionally, it resulted in a reduction of 0.8–1.4 points on the VAS scale, resulting in the acceptance of the second research hypothesis. This reduction appears to be associated with the lower amount of hydrogen peroxide available to penetrate the dental structure and reach the pulp chamber [34, 36], potentially triggering an inflammatory response [37], which participants clinically reported as sharp, spontaneous pain [6].

However, there are conflicting findings regarding the absolute risk and intensity of TS when compared to previous clinical studies [27, 30]. Differences in the composition of products containing 6% hydrogen peroxide help explain these discrepancies. For instance, in one study, a 6% hydrogen peroxide gel containing N‐TiO_2_ was light‐activated [27]. Light activation of N‐TiO_2_ enhances hydrogen peroxide decomposition and reactive oxygen species generation, which may improve bleaching efficacy and compensate for the lower peroxide concentration [27, 28]. However, this same mechanism may also increase hydrogen peroxide diffusion through dental tissues, potentially leading to greater TS, as previously observed [27].

On the other hand, similar results were observed when the present findings were compared to a previous study that used the same commercial brand and experimental design [23]. This reinforces the importance of well‐designed randomized clinical trials to guide clinicians in selecting the appropriate bleaching gel concentration, balancing whitening efficacy with the risk of adverse effects for each individual patient.

Although there is no consensus regarding the ideal hydrogen peroxide concentration for in‐office treatments that balances effective with minimal TS, the findings of this study provide evidence that reducing the bleaching gel concentration to 6% can significantly lower both the risk and intensity of TS [23]. However, achieving whitening results comparable to those obtained with higher concentrations may require additional bleaching sessions or the combination with at‐home bleaching in a combined approach.

Finally, some limitations of this study should be acknowledged. Although two concentrations were evaluated, only one commercial brand and a self‐mixing formulation of in‐office bleaching gel were tested. Therefore, the findings cannot be generalized to other products on the market. Future studies should assess a wider range of concentrations and commercial formulations with different pH values, compositions, and rheological properties, as well as alternative protocols varying the duration of sessions. Additionally, because of the substantial differences between in‐office and at‐home bleaching products, the results obtained with the 6% concentration in this study should not be extrapolated to at‐home protocols. This distinction is particularly relevant since hydrogen peroxide gels (e.g., 6%) and carbamide peroxide gels (e.g., 15%–16%) may contain similar levels of available hydrogen peroxide.

## 5 Conclusions

1. In‐office dental bleaching using both 6% and 35% hydrogen peroxide concentrations produced significant tooth color improvement; however, the outcomes were not equivalent. The 35% concentration achieves superior bleaching efficacy with the same application protocol.
2. The 6% hydrogen peroxide concentration significantly reduced the risk and intensity of TS, representing a safer clinical alternative for patients prone to postbleaching sensitivity. The 35% concentration was associated with a higher risk and greater intensity of TS than the lower concentration.

## Funding

This work was supported by the Conselho Nacional de Desenvolvimento Científico e Tecnológico (304444/2025‐1) and the Coordinação de Aperfeiçoamento de Pessoal de Nível Superior (001).

## Conflicts of Interest

The authors declare no conflicts of interest.

## Data Availability

The data that support the findings of this study are available from the corresponding author upon reasonable request.
